# Comparison of neurally adjusted ventilatory assist and synchronized intermittent mandatory ventilation in preterm infants after patent ductus arteriosus ligation: a retrospective study

**DOI:** 10.1186/s12887-024-04727-w

**Published:** 2024-04-27

**Authors:** Hui-Zi Lin, Yun-Feng Lin, Yi-Rong Zheng

**Affiliations:** 1 Department of Neonatology, College of Clinical Medicine for Obstetrics & Gynecology and Pediatrics, Fujian Medical University, Fujian Children’s Hospital (Fujian Branch of Shanghai Children’s Medical Center), Fuzhou, China; 2 Department of Neonatology, Fujian Maternity and Child Health Hospital, College of Clinical Medicine for Obstetrics & Gynecology and Pediatrics, Fujian Medical University, Fuzhou, China; 3 Department of Cardiac Surgery, College of Clinical Medicine for Obstetrics & Gynecology and Pediatrics, Fujian Medical University, Fujian Children’s Hospital (Fujian Branch of Shanghai Children’s Medical Center), Fuzhou, China; 4 Fujian Key Laboratory of Women and Children’s Critical Diseases Research, Fujian Women and Children’s Hospital, Fuzhou, China

**Keywords:** Patent ductus arteriosus, Neurally adjusted ventilatory assist, Cardiac surgery, Infant, Premature

## Abstract

**Objective:**

This study aimed to compare the efficacy of neurally adjusted ventilatory assist (NAVA) to synchronized intermittent mandatory ventilation (SIMV) in preterm infants requiring mechanical ventilation after patent ductus arteriosus (PDA) ligation.

**Methods:**

A retrospective analysis was conducted on intubated preterm infants who underwent PDA ligation at our hospital from July 2021 to January 2023. Infants were divided into NAVA or SIMV groups based on the ventilation mode after surgery.

**Results:**

Fifty preterm infants were included. During treatment, peak inspiratory pressure (PIP) and mean airway pressure (MAP) were lower with NAVA compared to SIMV (PIP: 19.1 ± 2.9 vs. 22.4 ± 3.6 cmH_2_O, *P* < 0.001; MAP: 9.1 ± 1.8 vs. 10.9 ± 2.7 cmH_2_O, *P* = 0.002). PaO_2_ and PaO_2_/FiO_2_ were higher with NAVA (PaO_2_: 94.0 ± 11.7 vs. 84.8 ± 15.8 mmHg, *P* = 0.031; PaO_2_/FiO_2_: 267 [220–322] vs. 232 [186–290] mmHg, *P* = 0.025). Less sedation was required with NAVA (midazolam: 1.5 ± 0.5 vs. 1.1 ± 0.3 μg/kg/min, *P* < 0.001).

**Conclusion:**

Compared to SIMV, early use of NAVA post PDA ligation in preterm infants was associated with decreased PIP and MAP. Early NAVA was also associated with reduced sedation needs and improved oxygenation. However, further studies are warranted to quantify the benefits of NAVA ventilation.

## Introduction

Patent ductus arteriosus (PDA) is a condition that frequently affects preterm infants, with an incidence of roughly 46% in those less than 32 weeks gestation, and exceeding 70% in infants less than 28 weeks gestation [1, 2]. The condition results from a shunt that directs blood from the aorta to the pulmonary artery, leading to pulmonary congestion and a reduction in systemic blood flow. This in turn results in decreased lung compliance and tissue perfusion [3]. If medical therapy is contraindicated or does not succeed in preterm infants with significant PDA, surgical ligation becomes a necessity. Many premature infants who undergo PDA ligation rely on mechanical ventilation. Consequently, managing their post-surgery respiratory needs is of paramount importance. A key concern is lung immaturity, which can intensify respiratory difficulties in premature infants. Neurally adjusted ventilatory assist (NAVA) is an emerging ventilation mode that could address this issue by detecting diaphragmatic electrical activity to synchronize support with spontaneous breathing [4]. Research indicates that NAVA is both feasible and safe for use in neonates, infants, and children with conditions such as acute respiratory distress syndrome, prematurity, and bronchiolitis [5–8]. However, there is limited experience in utilizing NAVA in children after congenital heart surgery. This study explores our experiences with NAVA following PDA ligation in preterm infants. We assessed the feasibility of NAVA versus SIMV after PDA ligation in preterm infants using ventilation parameters, oxygenation metrics, and sedation requirements as the basis for comparing the efficacy of the two modes in providing adequate ventilation and oxygenation.

## Materials and methods

### Patients and data collection

We retrospectively reviewed intubated preterm infants who had PDA ligation from July 2021 to January 2023 in the cardiac intensive care unit at Fujian Children’s Hospital. Our study was approved by the Institutional Ethics Committee (No.2022ETKLR12026), waiving consent.

Inclusion criteria were preterm infants who had PDA ligation and were on postoperative mechanical ventilation, either SIMV or NAVA. Exclusion criteria were residual PDA shunt, contraindications to EAdi catheter like gastrointestinal bleeding or surgery, or acute respiratory distress syndrome requiring extracorporeal membrane oxygenation after ligation.

In our hospital, pediatric cardiac surgeons decide whether to ligate a PDA based primarily on echocardiography (e.g. PDA size, blood flow pattern, signs of excess volume or poor organ perfusion) and clinical factors (e.g. level of cardiorespiratory support needed, evidence of end-organ damage like high creatinine, gestational and chronological age) [9]. We collected data on demographics and outcomes from electronic medical records.

### Ventilation strategies

All infants were intubated with a cuffed endotracheal tube. After PDA ligation, all infants received mechanical ventilation with a Servo-I ventilator (Maquet, Sweden) capable of delivering SIMV or NAVA, as chosen by the cardiac ICU attending. Initial SIMV settings were standardized for peak inspiratory pressure (PIP) 14–16 cmH_2_O, positive end-expiratory pressure (PEEP) 3–6 cmH_2_O, fraction of inspired oxygen (FiO_2_) 0.4–0.6 and then titrated according to saturations, respiratory rate (RR) 30–40 breaths/min. Ventilation aimed for PaCO_2_ 35–45 mmHg and PaO_2_ 50–80 mmHg.

For NAVA, pressure support ventilation levels were titrated over 5 min to target tidal volumes of 6–8 ml/kg and RR of 30–40 breaths/min. The initial FiO_2_ was generally set at 0.4–0.6 and titrated thereafter to target 90–95% oxygen saturation; PEEP was set between 3–6 cmH_2_O [10, 11]. Diaphragmatic electrical activity (EAdi) was measured using a NAVA catheter (Maquet, Sweden) with electrodes to detect phrenic nerve signals [12]. The Servo-I guided catheter positioning. NAVA can trigger inhalation to exhalation based on EAdi or airway dynamics using a “first-come, first-served” algorithm. NAVA levels were reassessed and adjusted based on EAdi, respiratory parameters and ventilation.

Extubation criteria were: hemodynamic stability, FiO_2_ ≤ 0.4, PIP ≤ 16 cmH_2_O, PEEP 2–4 cmH_2_O. The same team managed all patients. Sedation used midazolam (1–3 mcg/kg/min), adjusting as needed. The Neonatal Pain, Agitation and Sedation Scale (N-PASS) guided sedation to a score of -2 [13].

### Data collection

We retrospectively reviewed all medical records, including ventilator records. Baseline factors included: demographics; PDA size/body weight ratio; left atrium-to-aorta ratio; preoperative pulmonary hypertension; prior ibuprofen; cardiothoracic ratio; history of lung disease; preoperative oxygen index, neonatal respiratory distress syndrome and intraventricular hemorrhage; ventilator dependence; and duration of NAVA. We summarize the baseline demographic characteristics of the patients in Table 1.

**Table 1:** Demographic and baseline characteristics^a^

Characteristics	SIMV (*n* = 23)	NAVA (*n* = 27)	*P* Value
Sex (Male/Female)	13/10	12/15	0.395
GA at birth (weeks; mean ± SD)	28.8 ± 3.1	29.5 ± 2.6	0.381
BW at birth (gram; mean ± SD)	1295 ± 234	1313 ± 251	0.795
Age at surgery (days; mean ± SD)	23.1 ± 7.3	24.9 ± 7.5	0.385
BW at surgery (kg; mean ± SD)	1608 ± 290	1630 ± 322	0.802
LA/AO ratio (mean ± SD)	1.66 ± 0.10	1.69 ± 0.08	0.158
PDA size (mm)	2.7 ± 0.3	2.5 ± 0.2	0.104
Pre-op pulmonary hypertension, n (%)	17 (74)	21 (78)	0.712
Prior ibuprofen treatment, n (%)	21 (91)	23 (85)	0.674
Pre-op invasive ventilation, n (%)	20 (87)	25 (93)	0.651
Pre-op oxygen index (mean ± SD)	9.3 ± 4.0	8.9 ± 3.7	0.712
Pre-op PIP (cmH_2_O; mean ± SD)	20.8 ± 3.6	19.5 ± 3.3	0.202
Pre-op PEEP (cmH_2_O; mean ± SD)	4.8 ± 1.0	4.6 ± 1.1	0.513
Pre-op MAP (cmH_2_O; mean ± SD)	11.5 ± 3.0	10.7 ± 2.5	0.277
Pre-op FiO_2_, mean ± SD	0.35 ± 0.11	0.38 ± 0.13	0.369
Pre-op NRDS, n (%)	19 (83)	24 (89)	0.689
Pre-op IVH, n (%)	8 (35)	9 (33)	1.000

*GA* Gestational age, *BW* Body weight, *LA/AO* Left atrium-to-aorta, *PDA* Patent ductus arteriosus, *PIP* Peak inspiratory pressure, *PEEP* Positive end-expiratory pressure, *MAP* Mean airway pressure, *FiO*_*2*_ Fraction of inspired oxygen, *NRDS* Neonatal respiratory distress syndrome, *IVH* Intraventricular hemorrhage, *NAVA* Neurally adjusted ventilatory assist

^a^Data reported as number and percentage or mean ± standard deviation

The primary outcome was the feasibility of using NAVA (compared to SIMV) for post-operative ventilation following PDA ligation. This feasibility was evaluated by examining key ventilator parameters, including peak inspiratory pressure (PIP) and mean airway pressure (MAP). The secondary outcomes included vital sign stability (mean arterial pressure, respiratory rate, heart rate), oxygenation efficiency, sedation requirements (midazolam dose needed to maintain target sedation scale) and the absence of NAVA-related complications. Ventilator settings recorded were: tidal volume (VT), PIP, MAP and arterial blood gases (ABGs) (pH, PaO_2_, PaCO_2_, PaO_2_/FiO_2_, lactate) from the time NAVA or SIMV began. ABGs were done 30 min after starting NAVA/SIMV and repeated every 4–6 h or more often if needed. Patients were monitored using blood pressure measurements and pulse oximetry. Ventilator data were downloaded for analysis. Data were collected at 3-h intervals during the study period after PDA ligation.

### Statistical analysis

The sample size for this study was not predetermined. Instead, we included all eligible infants who met the study criteria. We used IBM SPSS software version 25.0 for Windows (IBM SPSS Inc., Chicago, IL, USA) to analyze the data. Continuous variables, which are presented as the mean ± standard deviation (SD), were analyzed using t-tests. Enumeration data are described using counts and percentages. To compare means, we employed Student’s t-test, while Fisher’s exact test was utilized for categorical data. For data that did not follow a normal distribution, we applied the Mann–Whitney U test. Furthermore, we performed multivariate linear regression analysis to control for potential baseline confounders. To analyze the repeated measurement data, we employed a linear mixed-effects model, which accounts for the correlation between repeated measurements within each patient and allows for the assessment of changes over time. We considered a two-sided *P*-value of less than 0.05 to indicate statistical significance.

## Results

### Patient characteristics

The characteristics of the included infants in both groups are presented in Table 1. A total of 50 preterm infants who were mechanically ventilated after PDA ligation were included in the study (23 in the SIMV group and 27 in the NAVA group). The baseline characteristics in the two groups are shown in Table 1. There was no significant difference in the demographics and preoperative baseline characteristics between the two groups of preterm infants (*P* > 0.05).

### Outcomes

Table 2 shows vital signs, ABGs and respiratory parameters based on ventilation mode. Using NAVA, PIP and MAP were significantly lower than with SIMV (PIP: 19.1 ± 2.9 vs. 22.4 ± 3.6 cmH_2_O, *P* < 0.001; MAP: 9.1 ± 1.8 vs. 10.9 ± 2.7 cmH2O, *P* = 0.002). After controlling for potential confounders through multivariate regression, the grouping factor retained a statistically significant association with both MAP (*P* = 0.001) and PIP (*P* = 0.001). PaO_2_ and PaO_2_/FiO_2_ were significantly higher with NAVA (PaO_2_: 94.0 ± 11.7 vs. 84.8 ± 15.8 mmHg, *P* = 0.031; PaO_2_/FiO_2_: 267 [220–322] vs. 232 [186–290] mmHg, *P* = 0.025). Midazolam doses were lower during NAVA (1.5 ± 0.5 vs. 1.1 ± 0.3 μg/kg/min, *P* < 0.001). There were no significant differences in mean arterial pressure, heart rate, FiO_2_, respiratory rate, tidal volume, pH, PaCO_2_ or lactate between ventilation modes. Table 3 shows complications and short-term outcomes, which did not significantly differ between groups (*P* > 0.05).

**Table 2:** Vital signs and laboratory values during the study period^a^

Variable	SIMV (*n* = 23)	NAVA (*n* = 27)	*P* Value
Heart rate, (beats/min; mean ± SD)	161 ± 3	162 ± 5	0.358
MBP, (mmHg; mean ± SD)	45 ± 3	44 ± 2	0.065
Breathing frequency, mean ± SD	45 ± 9	48 ± 7	0.272
FiO_2_, mean ± SD	0.39 ± 0.13	0.40 ± 0.14	0.803
VT (mL/kg; mean ± SD)	6.8 ± 1.8	6.3 ± 1.7	0.368
PIP (cmH_2_O; mean ± SD)	22.4 ± 3.6	19.1 ± 2.9	** < 0.001**
MAP (cmH_2_O; mean ± SD)	10.9 ± 2.7	9.1 ± 1.8	**0.002**
pH, mean ± SD	7.41 ± 0.03	7.41 ± 0.06	0.539
PaO_2_, (mmHg; mean ± SD)	85.3 ± 16.0	94.0 ± 11.7	**0.031**
PaCO_2_, (mmHg; mean ± SD)	48.1 ± 7.3	47.4 ± 6.5	0.924
PaO_2_/FiO_2_ ratio, median (IQR)	232 (186–290)	265 (220–322)	**0.025**
Lactates, (mmol/l; mean ± SD)	1.1 ± 0.5	1.2 ± 0.4	0.542
Midazolam (ug/kg.min; mean ± SD)	1.5 ± 0.5	1.1 ± 0.3	** < 0.001**

*Abbreviations: MBP* Mean arterial blood pressure, *VT* Tidal volume, *PIP* Peak inspiratory pressure, *MAP* Mean airway pressure, *FiO*_*2*_ Fraction of inspired oxygen, *PO*_*2*_ Partial pressure of oxygen, *PCO*_*2*_ Partial pressure of carbon dioxide

^a^Data reported as number and percentage or mean ± standard deviation or median (IQR)

**Table 3:** Complications and outcomes of the study infants

Variable	SIMV (*n* = 23)	NAVA (*n* = 27)
Post-op IVH, n (%)	3 (13)	4 (15)
PVL, n (%)	1 (4)	0
Post-op NEC, n (%)	3 (13)	2 (7)
VAP, n (%)	2 (9)	1 (4)
ROP, n (%)	4 (17)	3 (11)
BPD, n (%)	12 (52)	14 (52)
Post-op pulmonary hypertension, n (%)	6 (26)	6 (22)
Duration of post-op MV, d, median (IQR)	4 (2–12)	5 (4–15)
Complications of surgery	4 (17)	6 (22)
Pneumothorax, n (%)	2 (9)	3 (11)
Phrenic nerve palsy, n (%)	1 (4)	2 (7)
Vocal cord paralysis, n (%)	1 (4)	1 (4)
Hemothorax, n (%)	0	0
Death, n (%)	1 (4)	0

*IVH* Intraventricular hemorrhage, *PVL* Periventricular leukomalacia, *NEC* Necrotizing enterocolitis, *VAP* Ventilator associated pneumonia, *ROP* Retinopathy of prematurity, *BPD* Bronchopulmonary dysplasia, *MV* Mechanical ventilation

## Discussion

Infants after congenital heart surgery often require mechanical ventilation. However, recently, most infants after cardiac surgery are extubated in the operating room. The exception is patients with complex neonatal congenital heart disease or ligation of the ductus arteriosus in premature infants, which often require prolonged mechanical ventilation. These infants require not only additional respiratory assistance but also comprehensive hemodynamic management, including various inotropes, to sustain sufficient cardiac output and avert cardiopulmonary complications. Additionally, patent ductus arteriosus results in increased pulmonary blood flow and congestion. Coupled with lung immaturity in preterm infants, this congestion leads to a greater need for respiratory support [14]. Therefore, it is very important to optimize the ventilation strategy of the patient’s hemodynamics while avoiding unnecessary increases in medical support. in medical support [15].

At our CICU, it has become standard practice to introduce NAVA to patients following PDA ligation in preterm infants if patient-ventilator asynchrony affects hemodynamics. Because of the immature lung development of premature infants, they are more susceptible to ventilator-associated lung injury (VILI). VILI will aggravate the lung development disorder occurring after birth, causing serious sequelae such as bronchopulmonary dysplasia [16]. The optimal ventilation mode should provide adequate and steady tidal volume and minute ventilation at low airway pressure. It should also be able to rapidly adapt to sudden or unpredictable changes in lung mechanics or patient requirements. NAVA records the EAdi through the esophageal catheter, and the degree of assistance is determined by the patient’s breathing needs, synchronizing the ventilator’s breathing with the patient’s inhalation, thereby reducing the patient’s need for pressure [17]. Crulli et al. conducted a study in 28 children with congenital heart disease after surgery and found that the PIP and MAP were significantly reduced after NAVA respiratory support compared with conventional ventilation [18]. Liet et al. compared hemodynamic parameters in a physiology pilot study of 6 children and found that inspiratory pressure was lower and blood pressure was higher with NAVA compared with conventional ventilation [19]. In our study, a similar situation was observed. That is, the airway PIP and the MAP were significantly reduced compared with SIMV. Furthermore, we found that oxygenation under NAVA was improved over SIMV modes, which was similar to the results of the previous study [17]. Therefore, better oxygenation can be achieved with lower airway pressure under NAVA, which may help minimize ventilator-induced lung injury. This adds to the limited literature in this population.

Our study found that the sedation requirement during NAVA ventilation was significantly lower than that of SIMV ventilation mode. A randomized controlled study of 170 pediatric intensive care patients by Kallio et al. also found less sedation required with NAVA compared to conventional ventilation [20]. Lee et al. retrospectively analyzed NAVA and conventional ventilation in 14 ventilator-dependent preterm infants with bronchopulmonary dysplasia and found that sedation requirements were significantly reduced in the NAVA group [21]. SIMV mostly use pressure-triggered or flow-triggered, both of which have an inherent delay in-breath initiation compared to NAVA. Since the triggering of NAVA is driven by electrical signals from the diaphragm, synchronization of the patient with the ventilator is almost always present. This synchronization between the patient and the ventilator helps minimize patient discomfort and anxiety, with reduced sedation requirements following the administration of NAVA [5, 22].

To the best of our knowledge, this is the first study to document the use of NAVA following ductal ligation in premature babies. However, this study has some limitations. Firstly, since it was a retrospective study, some inherent biases may have arisen. When reviewing the information in the electronic medical record system, we found that the data available did not allow the assessment of patient-ventilator synchronization or work of breathing. Second, this study was performed in preterm infants following ductal ligation surgery, which may hinder the application of the current findings to other infants with congenital heart disease. Moreover, limitations including small sample size, brief observation timeframe, and absence of long-term follow-up could also impact the precision of the findings. Although a small retrospective study, the results suggest the value of NAVA for PDA ligation in preterm infants. Additionally, the results will offer some reference value for further prospective studies moving forward, and encourage the use of NAVA in the perioperative management of congenital heart defects. Other factors like congenital defects, medications, feeds, fluids, ventilator settings, sedation, and anesthesia were uncontrolled. Standardizing these variables in prospective studies is necessary to isolate NAVA’s effects after PDA closure. Moreover, it is important to note that the observed differences in PIP and MAP between the NAVA and SIMV groups may be partially attributed to the use of target tidal volume in the NAVA mode. In NAVA, PIP automatically adjusts according to the patient’s tidal volume, whereas in SIMV, PIP settings require manual adjustment by clinicians. This inherent difference in ventilation strategies could contribute to the observed variations in PIP and MAP between the two groups. However, the early evidence of benefit warrants future rigorous NAVA assessment in this cohort following ductal ligation, while accounting for additional factors, to allow definitive conclusions.

## Conclusions

The use of NAVA appears to be feasible in preterm infants after patent ductus arteriosus ligation. Compared to SIMV, early use of NAVA post PDA ligation in preterm infants was associated with decreased PIP and MAP. Early NAVA was also associated with reduced sedation needs and improved oxygenation. Further prospective studies should be conducted to fully evaluate the effects of NAVA in this vulnerable patient population.

## Data Availability

The datesets used or analyzed during the current study are available from the corresponding author on reasonable request.
